# Off‐isocentric VMAT technique for breast cancer: Effective dose reduction to organs at risk and its applicability based on patient anatomy

**DOI:** 10.1002/acm2.14237

**Published:** 2024-01-11

**Authors:** Igor Prokofev, Nidal Salim

**Affiliations:** ^1^ Department of Radiotherapy European Medical Center Moscow Russia

**Keywords:** breast, lymph nodes, radiotherapy, regression model, treatment planning, VMAT

## Abstract

**Purpose:**

This study aims to explore the off‐isocentric volumetric modulated arc therapy (offVMAT) technique for breast cancer and determine its applicability based on patient anatomical parameters.

**Methods:**

We retrospectively analyzed 44 breast cancer patients with varied lymph node involvement using different arc designs. Off‐isocentric techniques were benchmarked against previously published arc techniques: classic arcs (clVMAT), tangential arcs (tVMAT), and split arcs (spVMAT). During optimization, target coverage was made for all plans as close as possible to the criteria D99% > 95% and Dmax < 110% of the prescribed dose. A novel patient categorization, based on anatomical parameters (auxiliary structures) rather than lymph node involvement, is introduced. This categorization considers the volume of ipsilateral organs at risk (OARs) adjacent to the target. A binary regression model was developed on these anatomical parameters. It predicts the likelihood of offVMAT (*P*[offVMAT]) achieving better criteria.

**Results:**

Using the regression model, patients were divided into two groups: *P*(offVMAT) > 0.5 and *P*(offVMAT) < 0.5. For the *P*(offVMAT) > 0.5 group, most tVMAT plans are unable to achieve the clinical objectives. Comparing offVMAT with spVMAT, offVMAT exhibited better dose parameters for the heart (V20, V10, and D2 are 7.1, 2.4, and 1.5 times lower respectively), ipsilateral lung (V20, V10, V5 and the mean dose are 1.4, 1.3, 1.2, and 1.2 times lower respectively). The average doses to the contralateral side are consistent. In the *P*(offVMAT) < 0.5 group, the tVMAT technique showed increased doses at medium and high levels, yet reduced doses in contralateral OARs compared to spVMAT and offVMAT. spVMAT showed lower doses in the contralateral lung relative to the offVMAT technique, while clVMAT trailed in both groups. Validation of the model yielded a 90% accuracy rate.

**Conclusions:**

The new off‐isocentric breast planning technique effectively reduces doses to ipsilateral OARs, maintaining acceptable contralateral mean doses. This technique has an advantage over other techniques for patients with intricate anatomies. It is evaluated using anatomical parameters, which are also used to build binary regression model, which shows the dependence of anatomical parameters on whether offVMAT is preferred for individual patients. Also, such anatomical parameters provide a more objective and precise comparison between different planning techniques.

## 1 INTRODUCTION

In recent years, highly conformal radiation therapy techniques, such as volumetric modulated arc therapy (VMAT), have been introduced to achieve the required target dose coverage while ensuring adequate sparing of normal tissue, offering an alternative to the three‐dimensional conformal radiotherapy (3D‐CRT) technique. Over time, there has been a gradual evolution of VMAT techniques. Nowadays, a variety of VMAT techniques compete for prominence in breast cancer treatment, and the arc designs for VMAT planning have grown increasingly complex.

The initial classic VMAT (clVMAT) techniques proposed the use of 1−3 arcs spanning 200−240°, beginning from the patient's back. This arc arrangement is still frequently employed today.^1^, ^2^ These VMAT techniques significantly accelerated dose delivery compared to IMRT while maintaining a similar dose distribution.^1^, ^3^, ^4^, ^5^, ^6^, ^7^ Furthermore, VMAT reduces doses to organs at risk (OARs) at high levels, outperforming the 3D‐CRT technique, which remains the predominant approach for whole breast irradiation.^8^, ^9^ A downside to the standard VMAT techniques is their propensity to increase doses to OARs at low dose levels, and, as some authors note, to deliver high mean doses to ipsilateral OARs.^9^, ^10^, ^11^ These characteristics unfavorably differentiate classic VMAT from traditional techniques. Tangential VMAT (tVMAT) techniques have been widely adopted to address these low‐dose concerns.^10^, ^12^, ^13^, ^14^, ^15^ Some researchers have highlighted the superior dose distribution of tVMAT compared to 3D‐CRT, particularly regarding homogeneity and conformity indices for both localizations, irrespective of axillary lymph node involvement.^12^, ^14^, ^16^, ^17^, ^18^ Thus, tVMAT merges the advantages of 3D‐CRT's low dose levels at OARs with a superior planning target volume (PTV) dose conformity. However, tVMAT techniques amplify high doses to ipsilateral OARs due to a reduction in the number of beam directions (akin to the field‐in‐field technique) relative to clVMAT. This challenge is most pronounced in cases involving the axillary lymph and intramammary nodes, where tVMAT plans often prove clinically unsuitable.^12^, ^14^, ^16^, ^18^

The next important step in lymph node involving breast planning techniques was based on split arcs (spVMAT).^19^, ^20^ Researchers employed collimator for the best fit of PTV from different gantry angles. This method effectively reduced high dose levels to the ipsilateral lung and heart, delivering lower mean doses to the contralateral side in comparison to clVMAT. The underlying intent of the split field design was to guide the optimization process, achieved through the intermittent rotation of the collimator.

This article illuminates, for the first time, the advantages VMAT planning with an isocenter shifted relative to the center of mass of the PTV by more than 5 cm (offVMAT) and its artificial closure by the jaws are clearly demonstrated. A comparative analysis is presented between tVMAT, spVMAT, clVMAT, and the newly proposed offVMAT technique for breast treatment planning. This innovative method promises superior PTV coverage while maintaining minimal dose levels to ipsilateral OARs, especially beneficial for cases involving both axillary and intramammary (IM) lymph nodes.

Currently, there exists a spectrum of opinions regarding the superiority of one planning technique over another, even in breast‐ only treatment.^11^, ^21^, ^22^, ^23^, ^24^, ^25^, ^26^, ^27^ Multiple factors, ranging from the specific equipment in use and the experience of the physicist to the field arrangements, optimization process, and individual patient anatomies, influence the outcomes. However, by introducing parameters that delve into the nuanced differences between patients, we can mitigate the variance introduced by patient anatomy in treatment results. These parameters serve dual purposes: they enable a more precise comparison of patient groups with similar attributes across different studies and assist in selecting the most suitable planning technique for an individual. In this study, our aim is twofold: to introduce anatomical parameters offering a richer understanding of the patient demographics, and to present a binary regression model that elucidates how these anatomical parameters influence the success of the offVMAT technique in achieving optimal criteria for individual patients.

## 2 MATERIALS AND METHODS

### 2.1 Patient selection

A total of 44 left‐sided breast cancer patients with varying levels of lymph node involvement were retrospectively and randomly selected for this study. This included 33 patients with an intact breast, three with breast implants, and eight with chest walls.

To train the regression model, 24 patients were chosen:

- without any lymph node involvement (*n* = 8)
- with varied lymph node involvement, excluding IM nodes (*n* = 8)
- with comprehensive lymph node involvement, including I‐IV axilla levels and IM nodes (*n* = 8)

For validating the regression model, an additional 20 left‐sided breast cancer patients were selected as follows:

- without any lymph node involvement (*n* = 5)
- with varied lymph node involvement, excluding IM nodes (*n* = 10)
- with comprehensive lymph node involvement, encompassing I‐IV axilla levels and IM nodes (*n* = 5)

Patients were positioned head‐first in a supine position during the CT simulation, using the Q‐fix breast board with their arms raised above their heads. CT acquisition was with 2‐mm‐thick adjacent slices in free‐breathing mode, without employing the deep inspiration breath hold (DIBH).

The assessment involved seven physicians and four medical physicists from the radiotherapy department. They were tasked with choosing the optimal plan for each patient (from both the training and validation groups) from among four plans based on different techniques. The reviewers were kept blind to the patients' anatomical parameters and the planning techniques, seeing only the dose distribution for an unbiased evaluation. The review began with a collective assessment of four plans to standardize the review process and normalize Likert scale. This scale, ranging from 1 (unusable plan) to 4 (perfect plan), was utilized to evaluate the plans (as detailed in Table 1). When required, plans were juxtaposed for a clearer comparison. Likert scale scores from all reviewers were summed up for each plan, across the four techniques in both the training and validation sets. The plan garnering the highest cumulative score was deemed the best for that specific patient.

**TABLE 1:** Likert scale to score radiotherapy plans.

Score	Description
4	A perfect plan without the need for any changes.
3	Minor edits that are not necessary. Stylistic changes preferred, but not clinically important. Current plans are clinically acceptable.
2	Major edits. One of the constraints is not satisfied.
1	Major edits. Two or more of the constraints is not satisfied.

For all 44 patients, a radiation oncologist delineated the clinical target volume (CTV), adhering to the guidelines set forth breast contouring atlas by the Radiation Therapy Oncology Group (RTOG). To form the planning target volume (PTV_out_), a 3‐mm margin was added to the clinical target volume. The PTV_out_ was subsequently cropped 5 mm inside the patient's body contour, resulting in the structure termed as PTV.

During the optimization phase, a 5 mm water‐equivalent bolus was added near the boundary of the body contour during the optimization to take into account possible movements of the breast during treatment.^28^ The PTV_out_ was used as the target volume when optimizing VMAT plans. However, the final dose calculation and plan evaluations were conducted without this optimization bolus. The average volume of PTV_out_ was 1156  ± 558 cm^3^.

OARs identified in this study included the heart, contralateral and ipsilateral lungs, and the contralateral breast. Their respective average volumes are as follows: lung –1212 ± 320 cm^3^, heart –501 ± 132 cm^3^, contralateral breast –1095 ± 523 cm^3^, and contralateral lung –1344 ± 329 cm^3^). During the planning, the structures of the liver, esophagus, humeral head, and thyroid were taken into account.

### 2.2 Patient‐specific supporting volumes and anatomical parameters

**FIGURE 1:**
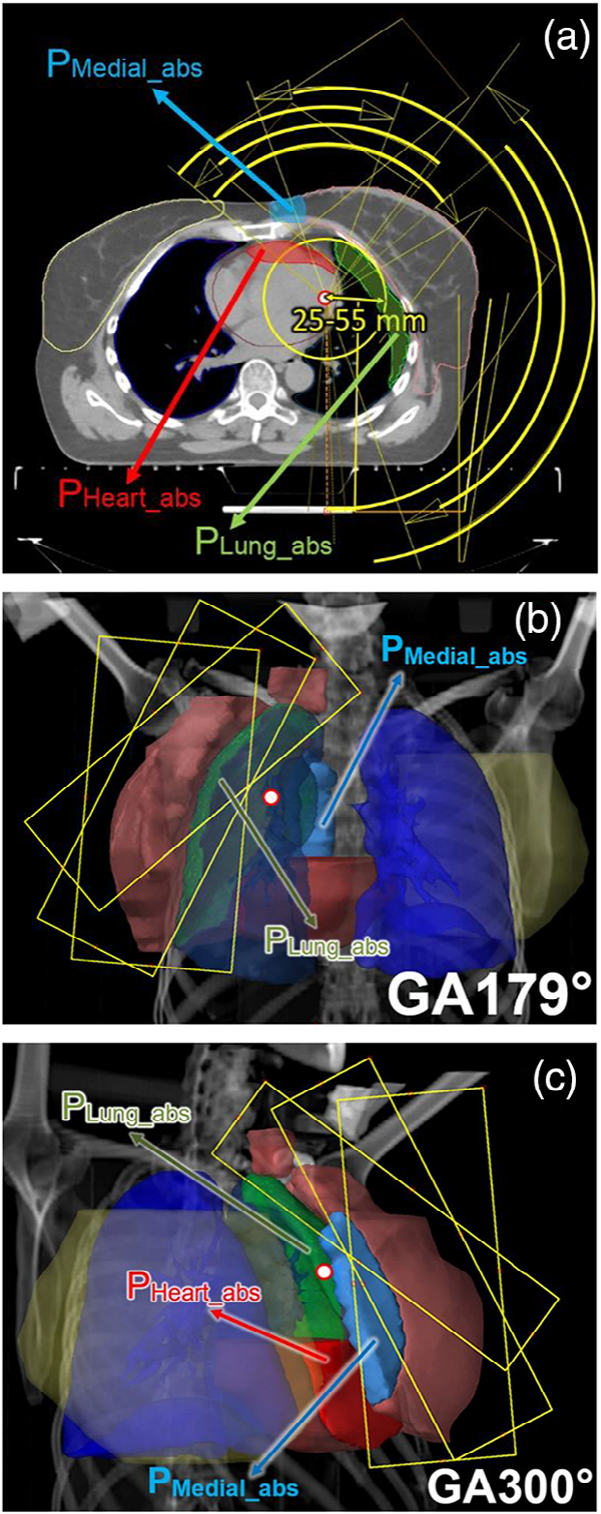
Isocenter positioning on an axial plane and supporting structures (a); off‐isocenter arc design beam eye of views (BEVs) from two different gantry angles (GAs): GA = 179° (b) and GA = 300° (c), illustrating the selection of collimator angles based on PTV shape.

This article introduces the novel concept of anatomical parameters, offering a more intricate depiction of patient populations. These parameters highlight the proportion of a critical organ's volume situated near the PTV. They prove more insightful than mere information about lymph nodes encompassed within the PTV because they can also serve as numerical predictors for selecting the optimal planning technique. Notably, patients can exhibit similar anatomical parameters even with diverse lymph node involvement. For the calculation of these anatomical parameters, supporting volumes were delineated (Figure 1).

To evaluate the position of ipsilateral OARs in relation to the PTV, two supporting volumes for the heart and ipsilateral lung (*P*_Heart_abs_ and *P*_Lung_abs_) were created. These are situated 3 and 2 cm away from the PTV, respectively. For this purpose, ring structures with an outer boundary of 3 cm or 2 cm and an inner boundary of 1 cm were formed around the PTV. Intersections of these rings with the heart or ipsilateral lung volumes were created using the Boolean operator ‘AND’ (see Figure 1). An additional supporting volume was generated by forming a 5 cm ring structure around the contralateral breast. The resulting structure, termed *P*_Medial_abs_, was created by intersecting this 5‐cm ring with the PTV using the Boolean operator ‘AND’ (as depicted in Figure 1). This particular volume was selected given the challenges in achieving dose coverage of the PTV adjacent to the sternum.

(1) $$\begin{array}{ccc} P_{\mathrm{Heart}} & = & \frac{V\left(P_{\mathrm{Heart}\_\mathrm{abs}}\right)\left(\mathrm{cc}\right)}{V\left(\mathrm{Heart}\right)\left(\mathrm{cc}\right)};\\ P_{\mathrm{Lung}} & = & \frac{V\left(P_{\mathrm{Lung}\_\mathrm{abs}}\right)\left(\mathrm{cc}\right)}{V\left(\mathrm{Ipsilateral}\mathrm{Lung}\right)\left(\mathrm{cc}\right)};\\ P_{\mathrm{Medial}} & = & \frac{V\left(P_{\mathrm{Medial}\_\mathrm{abs}}\right)\left(\mathrm{cc}\right)}{V\left(\mathrm{PTV}\right)\left(\mathrm{cc}\right)}, \end{array}$$

The *P*_Heart_ anatomical parameter is determined by the ratio of *P*_Heart_abs_ volume to the overall heart volume, while the *P*_Lung_ anatomical parameter is calculated as the ratio of *P*_Lung_abs_ to the volume of the ipsilateral lung. Both *P*_Heart_ and *P*_Lung_ represent the proportion of heart or ipsilateral lung volume situated near the PTV. The *P*_Medial_ anatomical parameter is the quotient of *P*_Medial_abs_ volume to the PTV volume (as represented in Equation 1).

### 2.3 Treatment planning

VMAT plans were optimized in the Eclipse treatment planning system (Varian Medical Systems, Palo Alto, CA) using a TrueBeam linear accelerator, utilizing 6 MV photons. The accelerator is equipped with a millennium 120 multileaf collimator (MLC) with jaw tracking option, which was employed across all plans. Plans were optimized using the Photon Optimizer algorithm (version 15.6) and calculated with Acuros XB (version 15.6) with 0.2 cm grid size. The aperture shape controller strength was set to “moderate” during optimization that tends to increase the size and decrease the complexity of the MLC aperture.

The prescription dose to the breast/chest wall was 2.67 Gy in 15 fractions. The planning aim was to achieve at least 99% of the volume of the PTV receiving 38.05 Gy (i.e., the 95% of the prescription dose) and the mean heart dose <5 Gy, V20Gy < 5%; the ipsilateral lung dose V20Gy < 15%, V10Gy < 30%, and V5Gy < 50%, and mean dose <10 Gy; contralateral lung dose V5Gy < 10% while keeping the mean doses at the contralateral breast as low as possible.

Four distinct techniques were applied to each patient: offVMAT, clVMAT, tVMAT, and spVMAT. This resulted in 96 plans for the training set and 80 for the validation set. All plans were planned by one person. During the optimization process, emphasis was placed on reducing the mean doses to the heart and ipsilateral lung and on enhancing the dose uniformity within the target. A ring structure around the PTV with an outer boundary of 2.5 cm and an indentation of 0.5 cm from the PTV was used to minimize 95% prescribed dose outside the target volume to improve the dose distributions and the dose conformity to the target. Identical constraints, weights, and planning strategies were consistently used during the optimization of each VMAT technique for all patients. During the optimization, the weights assigned to the OARs were gradually increased, starting at roughly one‐third of the PTV weight and concluding at two‐thirds. Typically, two to three optimization iterations were conducted for each plan to refine PTV coverage. The “on” setting for the mode convergence value was selected during the optimization.

### 2.4 Isocenter, arc, and collimator arrangement

**FIGURE 2:**
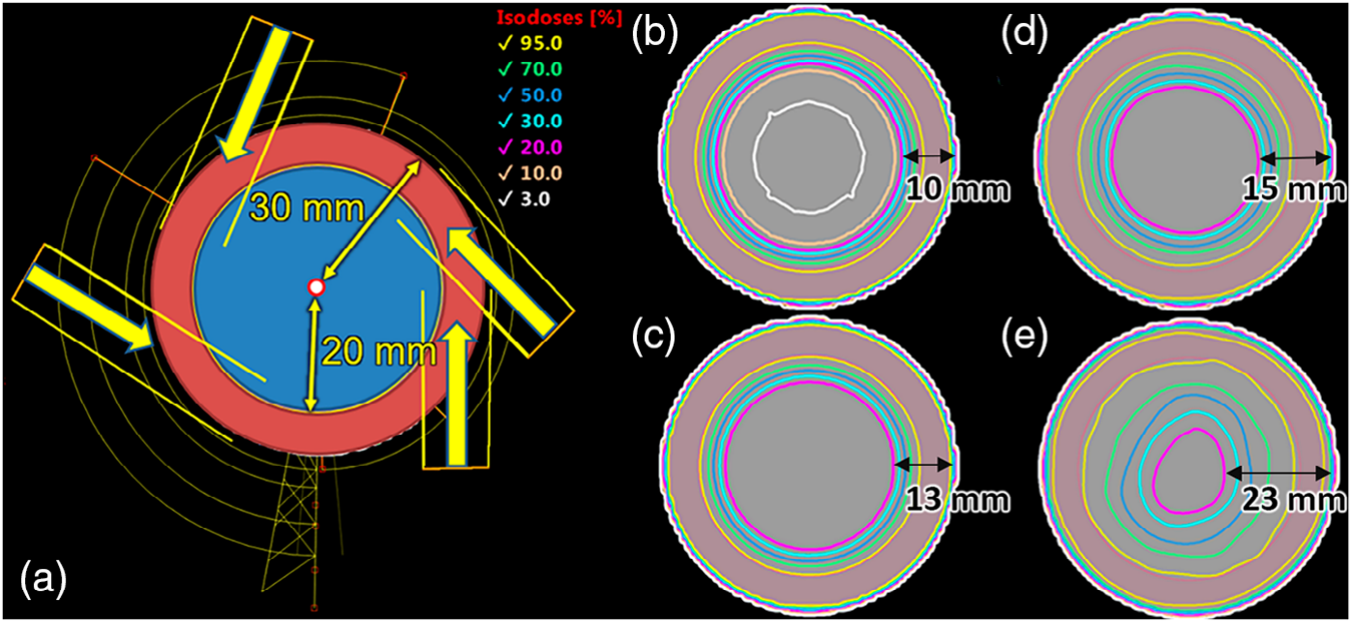
The irradiation of a phantom layer (red) close to the surface with protection for the coaxial cylinder (blue) using jaws. When the isocenter is positioned outside the primary beam at every gantry angle in the cylinder's center, the primary beam “avoids” the blue cylinder (a); isodose distribution on transverse views of jaw opening geometry off‐isocentric volumetric modulated arc therapy (offVMAT) technique (b); multileaf collimator (MLC) opening geometry without modulation (c); volumetric modulated arc therapy (VMAT) dose distribution with using avoid (entry + exit tool) structure in center phantom during optimization (d); classic (VMAT) technique (e).

To elucidate the advantages of this method, consider a simple model: a cylindrical phantom, which illuminates the idea behind the new technique (Figure 2a). Assume that we need to irradiate the layer of the phantom close to its surface (colored red) while minimizing the dose to the central coaxial cylinder (colored blue).

Classically during VMAT treatment planning isocenter is placed at or near the PTV's center of mass. Therefore, collimator aperture is opened to lungs and heart through the PTV from most of the gantry angles. Implemented treatment planning technique offVMAT suggests isocenter placement on the axis of the cylinder (Figure 2a). At the same time jaws protect coaxial cylinder volume (blue) while gantry moving around and beaming PTV layer on its surface. At any time, primary beam does not «see» coaxial cylinder. In this case, it is possible to irradiate any point on the surface of the cylinder (red) by rotating the gantry around the isocenter. This approach allows to achieve sharp dose gradient PTV to OARs, because at any given point beam is not open to OAR, but rather tangentially to it.

Dose distribution with jaw only opening geometry (offVMAT technique) is shown in Figure 2b. The case of using MLC without modulation instead of jaws with the same geometry is demonstrated in Figure 2c. Isodose level 20% is located deeper than jaw design: 10 mm versus 13 mm. Also, isodose levels 10% and 3% are not identified in the MLC design in the center of the phantom. Figure 2d shows VMAT dose distribution with using avoid (entry‐exit tool) structure in center phantom during optimization. Isodose level 20% is located at a depth of 15 mm. Figure 2e demonstrated classic VMAT technique with isodose level 20% identified at average depth—23 mm. Isodose levels 10% and 3% are also missing in avoid and classic VMAT design in the center of the phantom. Even if the part of the primary beam directed to the blue cylinder is closed with the leafs, transmission is the reason of the presence of a low‐dose bath when using classic VMAT. On the other hand, jaw‐only opening geometry provides the maximum gradient from the surface to the center of the phantom.

However, in the case of real‐world breast irradiation, the situation is complicated by a non‐cylindrical breast and the ipsilateral lung shapes, hot spots dose constraint for the breast, the inability to deliver the dose from the contralateral side of the body due to the hands of the patient, and the strict dose constraints for contralateral OARs. In addition, the maximum distance the collimator jaw can be shifted over the central axis is 2 cm for the X jaws on a Varian machine. These factors impose restrictions on the distance between the isocenter and PTV. Therefore, it is only possible to minimize the number of gantry angles from which the primary beam is directed into the ipsilateral OARs. Thus, it is necessary to use different collimator angles with varying arc lengths.

offVMAT planning success is largely determined by the isocenter position. It does not have definite position even in a specific patient. In general, the farther the isocenter is located from the PTV, the less ipsilateral OARs exposed, but the same time more dose to patient contralateral side and vice versa. Therefore, isocenter location is determined by both patient geometry and clinical objectives. First, axial plane with the most problematic area during planning was determined, where the dose gradient from the PTV to the ipsilateral organs would be the most difficult to create. The isocenter was located near this plane (Figure 1a). Typically, this is the plane with the largest *P*_Heart_abs_ or *P*_Lung_abs_ area and/or the largest edge curvature that is close to the PTV. Further, the isocenter was moved 25–55 mm toward the heart/lung at the axial plane from the PTV (Figure 1a). To do this, it is convenient to use the circle cursor tool in Eclipse with the appropriate radius. Note that the middle position between the PTV edges was not chosen always. Sometimes the best criteria achievement was obtained while the isocenter was placed closer to the patient's most problematic area, for example, near the largest *P*_Heart_abs_ volume (if it is clinically justified to protect the heart as much as possible). Then, in the frontal plane, the isocenter was shifted along the craniocaudal axis with an indent of 30–50 mm from the top of the lung using the BEV (beam eye of view) tool (Figure 1b). Generally, for patients whose PTV does not include supraclavicular lymph nodes, the isocenter position along the craniocaudal axis does not play a significant role.

The arc arrangement used six partial arcs in offVMAT plans with the gantry running: three back arcs 160–40°, 35−179°, and 179−340° and collimator rotation 320°, 340°, and 355° respectively and three contralateral side arcs 300−40°, 60−300°, and 300−20° and collimator rotation 5°, 25°, and 45° respectively. Arc and collimator angles are slightly (± 10−20°) vary depending on individual patient geometry. Ipsilateral lung side X jaws extend over the central axis for 2 cm for all arcs (Figure 1b,c). The collimator angles for all arcs were chosen in such a way that viewing in arc motion using the BEV, the maximum volume of PTV was located inside the irradiated field while capturing the minimum volume of the ipsilateral lung/heart for all gantry angles. It is critically important to ensure that each part of the target volume remains inside of the collimator aperture for a sufficient number of angles. An XML template of the resulting plan is available in Supplementary Materials (Supplementary Materials S01).

The clVMAT technique used three arcs with 179−300° gantry angles and three collimator rotations –350°, 10°, and 15°.^1^ The tVMAT technique used six partial arcs: three arcs with 179–105° gantry angle and three arcs with 300–0° gantry angle. The rotations of the collimator were set in accordance with the anatomical characteristics of the patients with jaws width no more than 15–18 cm.^15^ The spVMAT technique was used as recommended by the authors.^20^ VMAT design having five subarcs: two on contra side, two on back side and one 300°−60° with collimator angle 80°−100° to avoid heart. The isocenter was moved 2 cm toward the lung at the axial plane. Collimator angles for subarcs was set to ±10° with slight individual adjustments with PTV shape.

### 2.5 Quality assurance and data analysis

All plans underwent an evaluation using dose‐volume histogram metrics. The dose conformity index (CI) and homogeneity index (HI) were calculated for every plan (2).

(2) $$\begin{array}{ccc} & & \mathrm{CI}=\frac{V\;95\left(\mathrm{PTV}\right)\left[\mathrm{cc}\right]}{V\left(\mathrm{PTV}\right)\left[\mathrm{cc}\right]}\frac{V\;95\left(\mathrm{PTV}\right)\left[\mathrm{cc}\right]}{V\;95\left[\mathrm{cc}\right]};\\ & & \mathrm{HI}=\frac{D2\%\left(\mathrm{PTV}\right)-D98\%\left(\mathrm{PTV}\right)}{D50\left(\mathrm{PTV}\right)}, \end{array}$$

where V95(PTV)(cc) and V95(cc) are the PTV and whole body volumes, respectively, receiving 95% or more of the prescribed dose, and V(PTV)(cc) is the volume of PTV. CI ≤ 1, higher value indicating better conformity. The HI was calculated as (2) where DX%(PTV) (Gy) is the dose received by X% of the volume of PTV, and D50(PTV) (Gy) is the median dose of the PTV. Lower HI values suggest better dose homogeneity.

For the OARs, the mean doses, V5Gy, V10Gy, and V20Gy were compared for the left lung; the mean doses, V5Gy, V10Gy, and V20Gy, D2% were compared for the heart; mean doses, V5Gy and D2% for contralateral breast; mean doses, V10Gy and V5Gy were compared for the contralateral lung. Paired samples *t*‐test following a normality test (Shapiro–Wilk) was used to compare the results. Statistical significance was considered when *p* <0.05.

Patient‐specific quality assurance (QA) for the offVMAT plans was performed using the using ArcCHECK (SunNuclear). The results were analyzed according to the gamma evaluation using 3% as the dose difference and 2 mm as the distance to the agreement with a 10% threshold. The global gamma passing rate should be ≥95%.

In this study, we developed a logistic binomial regression model to ascertain the suitability of the offVMAT technique for individual patients based on their anatomical attributes. MATLAB (2022A, The MathWorks, Natick, MA, USA) facilitated all analyses. *P*_Heart_, *P*_Lung_, and *P*_Medial_ functioned as independent variables.

The model expression is as follows:

(3) $$\begin{array}{ccc} & & P\left(\mathrm{offVMAT}\right)=\\ & & \frac{1}{1+\exp(B0+B1\times P_{\mathrm{Heart}}+B2\times P_{\mathrm{Lung}}+B3\times P_{\mathrm{Medial}})}, \end{array}$$

where *B*0, *B*1, *B*2, and *B*3 logistic regression coefficients, *P*_Heart_, *P*_Lung,_ and *P*_Medial_ are anatomical parameters.

The MATLAB function “fitglm” was used to fit the model. The resulting model predicts the likelihood that a technique (either *P*(offVMAT) > 0.5 or *P*(offVMAT) < 0.5) will achieve better dose criteria, based on the anatomical parameters of the patient. The *p*‐values  < 0.05 indicate that the coefficients of the variables are statistically significant. The model was tested on a validation set consisting of 20 patients, reserved exclusively for this purpose.

## 3 RESULTS

### 3.1 Anatomical parameters and logistic regression model

From the training set, which included 24 patients and a total of 96 plans, the technique that garnered the highest aggregate Likert score was selected for each patient: 4 spVMAT, 6 tVMAT, and 14 offVMAT. Similarly, for the validation set of 20 patients (and a total of 80 plans), the distribution was: four spVMAT, seven tVMAT, and nine offVMAT. Notably, clVMAT was never selected as the superior plan in either set.

The involvement of multiple lymph nodes doesn't necessarily equate to high values of the anatomical parameters. This varies depending on the individual's anatomical specifics. For instance, a patient with anatomical parameters of *P*_Heart_ –0.2, *P*_Medial_ –0.07, and *P*_Lung_ –0.24 had no involved lymph nodes, and the parameters pertained solely to the breast. Yet another patient, with all lymph nodes involved, exhibited similar parameters: *P*_Heart_ – 0.2, *P*_Medial_ – 0.09, and *P*_Lung_ – 0.23.

**FIGURE 3:**
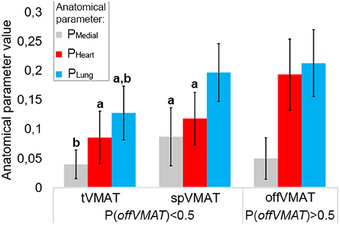
Distribution of anatomical parameters *P*_Heart_, *P*_Lung_, and *P*_Medial_ for all 44 patients depending on plans with maximum Likert score. ^a^Statistically significant difference (*p* < 0.05) in pairwise comparison against offVMAT technique. ^b^Statistically significant difference (*p* < 0.05) in comparison tangential volumetric modulated arc therapy (tVMAT) against split volumetric modulated arc therapy (spVMAT) technique.

The distribution of the *P*_Heart_, *P*_Lung_, and *P*_Medial_ parameters, based on plans with the topmost Likert score for all patients (from both training and validation sets), is depicted in Figure 3. The average values (and their standard deviations) for the full patient set are: *P*_Heart_ –0.16 ± 0.08, *P*_Lung_ –0.20 ± 0.07, and *P*_Medial_ –0.06 ± 0.04. In the training set, these metrics are *P*_Heart_ –0.18 ± 0.08, *P*_Lung_ –0.20 ± 0.08, and *P*_Medial_ – 0.08 ± 0.04. The values exhibit heterogeneity, which underscores the advantages of each technique given different anatomical parameters.

A distinct correlation emerges between anatomical parameters and the favored planning technique. As the *P*_Heart_ value rises, the more preferable offVMAT technique than tVMAT and spVMAT (Figure 3) across all patients. The average *P*_Heart_ scores for tVMAT and spVMAT plans stood at 0.09 ± 0.05 (*p* < 0.01) and 0.13 ± 0.05 (*p* < 0.01) respectively, whereas for offVMAT it was 0.21 ± 0.07. The average *P*_Lung_ for tVMAT and spVMAT were 0.14 ± 0.06 (*p* < 0.01) and 0.21 ± 0.04 respectively, and for offVMAT it was 0.23 ± 0.06. The *P*_Medial_ averages for tVMAT, spVMAT, and offVMAT plans were 0.04 ± 0.03 (*p* < 0.01), 0.09 ± 0.05 (*p* = 0.03), and 0.05 ± 0.04 respectively. The clVMAT technique has been excluded from Figure 3 since it didn't obtain the highest Likert score even once.

When the volume of ipsilateral organs adjacent to the PTV increases, the feasibility of using the tVMAT technique (and to a lesser extent spVMAT) diminishes—mainly due to challenges in adhering to heart and ipsilateral lung dose constraints. When *P*_Heart_ values exceed 0.20, the offVMAT technique exclusively becomes the preferred choice. Similarly, with *P*_Heart_ values at or above 0.15 and *P*_Lung_ values at or above 0.25 simultaneous, offVMAT is prioritized. High *P*_Medial_ values combined with moderate *P*_Heart_ values tend to favor the spVMAT technique.

This analysis helps identify correlations between anatomical parameters and the optimal technique. Logistic regression model, based on patient anatomical parameters, achieved a perfect 100% sensitivity and specificity on the training dataset of 24 patients. The parameters of the model are likely to deteriorate with an increase in the sample of patients and plans. Also, because of the small sample size (especially in spVMAT technique) binary logistic regression was used rather than multinomial regression.

This model aligns perfectly with the technique choices made by the radiotherapy department for each patient. Key parameters of the model coefficients can be found in Table 2.

**TABLE 2:** Parameters of the logistic regression model coefficients for evaluating the choice of off‐isocentric volumetric modulated arc therapy (offVMAT) technique.

	*β*	SE	*t*	*p*	95%CI lower	95%CI upper
*B*0	35.8	4.5	8.0	<0.0001	45.2	26.5
*B*1	−345.0	45.3	−7.6	0.0001	−250.5	−439.5
*B*2	−66.2	13.2	−5.0	0.0001	−38.5	−93.9
*B*3	430.2	63.9	6.7	<0.0001	563.5	296.9

*Note*: β are the values logistic regression coefficients *B*0, *B*1, *B*2 and *B*3, Standard errors of the coefficient estimate β (SE), the *t*‐test for β (*t*), *p*‐values for β, and the 95% confidence intervals for coefficients β (95% CI).

The resulting model (4) denotes the dependence of anatomical parameters on the likelihood that offVMAT will achieve superior Likert scale scores among all VMAT techniques for the 24‐patient training sample, as a logit regression function when planning an individual patient.

(4) $$P\left(\mathrm{offVMAT}\right)=\frac{1}{1+\exp\left(35.8-345\times P_{\mathrm{Heart}}-66.2\times P_{\mathrm{Lung}}+430.2\times P_{\mathrm{Medial}}\right)},$$

where *P*_Heart,_ *P*_Lung_, and *P_Medial_*—the patient's anatomical parameters, and *P*(offVMAT) denotes the probability of the offVMAT technique achieving the best criteria among the techniques evaluated.

A value of *P*(offVMAT) closer to 1 suggests the offVMAT technique as preferred, while a value closer to 0 suggests otherwise.

Validation of the model on 20 patients from the validation set yielded a 90% accuracy rate. The model attained an 89% sensitivity and a 91% specificity with this validation dataset, with two plans showing a false positive and false negative result respectively. Higher sensitivity and specificity figures confirm the model's robustness and fit to the data.

### 3.2 PTV coverage

V95_PTV_(%) of the planning target volume (PTV) was consistent across various planning techniques, as shown in Table 3. Among these, the tVMAT plans had the highest dose maxima. When evaluating PTV coverage metrics such as homogeneity Index (HI), conformity Index (CI), dose minima, and maxima, spVMAT, and offVMAT designs showcased superior results compared to tVMAT and clVMAT plans.

**TABLE 3:** Dose‐volume histogram parameters for the PTV and number of monitor units of plans.

	offVMAT	spVMAT	clVMAT	tVMAT
V95%_PTV_(%)	99.2 ± 0.4	99.0 ± 1.4^a^	98.6 ± 1.3^a^	98.8 ± 0.7^a^
V110%_PTV_(%)	0.6 ± 1.1	1.0 ± 1.6	1.8 ± 2.4^a^	2.8 ± 2.9^a^
D_median_	41.2 ± 0.5	41.4 ± 0.5	41.6 ± 0.6^a^	41.7 ± 0.6^a^
HI	0.10 ± 0.01	0.11 ± 0.02	0.11 ± 0.02^a^	0.13 ± 0.02^a^
CI_paddick_	0.99 ± 0.05	0.98 ± 0.06	0.98 ± 0.09	0.88 ± 0.07^a^
MU	1650 ± 502	1261 ± 365^a^	1141 ± 335^a^	1096 ± 327^a^

Abbreviations: Cl, confirmity index; clVMAT, classic volumetric modulated arc therapy; HI, homogeneity index; MU, monitor unit; offVMAT, off‐isocentric volumetric modulated arc therapy; spVMAT, split volumetric modulated arc therapy; tVMAT, tangential volumetric modulated arc therapy.

^a^Statistically significant difference (*p* < 0.05) in pairwise comparison against offVMAT technique.

There wasn't a significant difference in PTV coverage between techniques. During the optimization phase, weight coefficients were assigned to ensure PTV coverage remained close to D99% >95% and to minimize areas receiving doses exceeding 110% of the prescribed dose. Such a strategy allows for a comparative assessment based primarily on the achievement OAR constraints. However, tVMAT (and to a lesser extent, clVMAT) did not meet the same HI, CIpaddick, and V110% PTV values as offVMAT and spVMAT. Consequently, a balance was sought between optimal PTV coverage and OAR constraint satisfaction.

Table 3 shows the average monitor units (MUs) used per fraction for each planning technique. The offVMAT technique required the highest number of MUs, while tVMAT used the fewest.

### 3.3 Organs at risk

**FIGURE 4:**
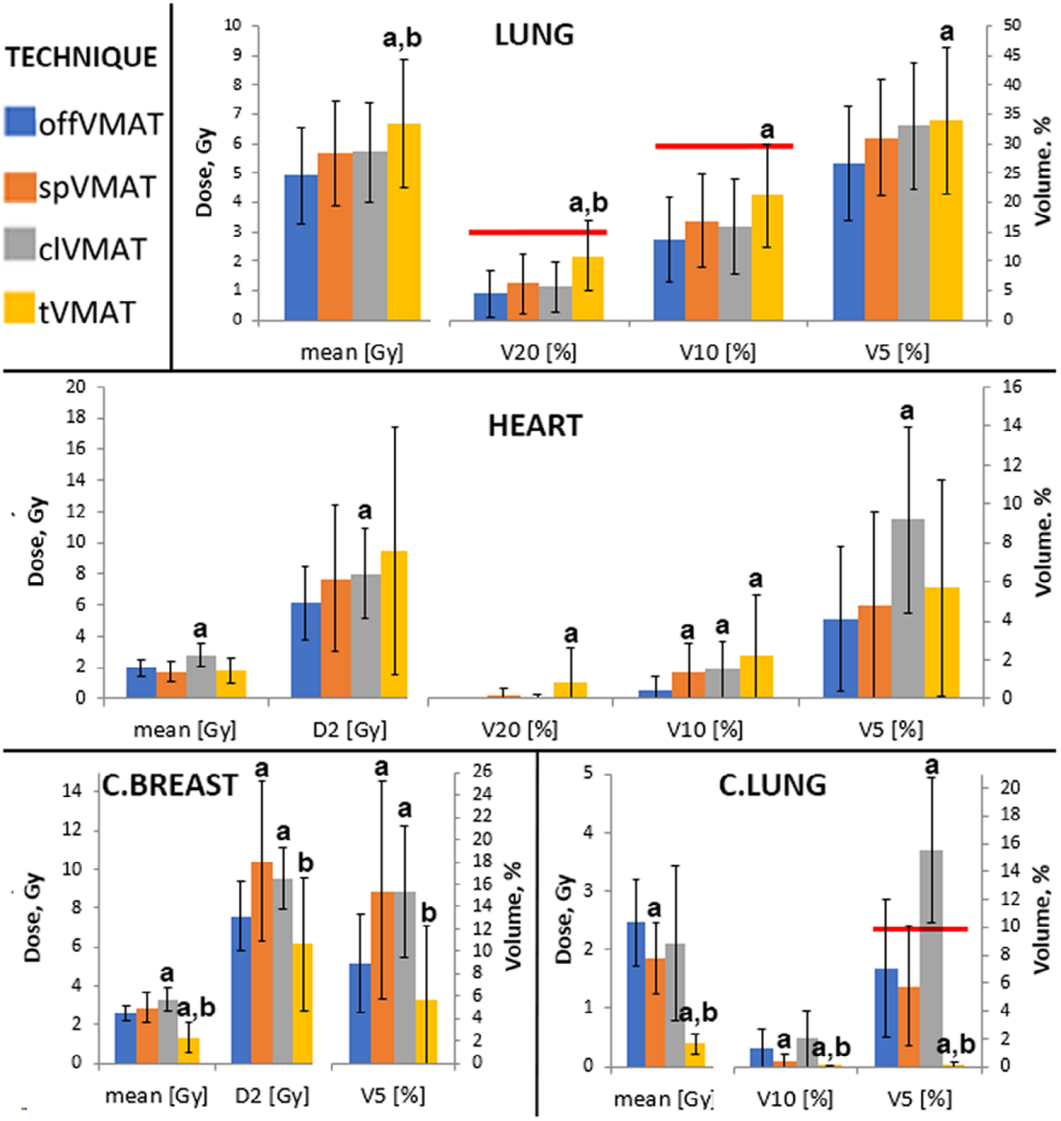
Dose parameters of organs at risk (OARs) (Mean ± SD) for patient group with *P*(offVMAT) < 0.5 parameter (off‐isocentric volumetric modulated arc therapy [offVMAT], split volumetric modulated arc therapy [spVMAT], classic volumetric modulated arc therapy [clVMAT], and tangential volumetric modulated arc therapy [tVMAT]). Red lines show the OAR constraint. ^a^Statistically significant difference (*p* < 0.05) in pairwise comparison against offVMAT technique. ^b^Statistically significant difference (*p* < 0.05) in comparison tangential volumetric modulated arc therapy (tVMAT) against split volumetric modulated arc therapy (spVMAT) technique.

It is difficult to reveal the advantages of one technique over another, when dose parameters statistics for the OARs are provided for the entire patient population. The values of the anatomical parameters, as well as the outcome of the model function (4), play a determining role in highlighting the advantages of one technique over another. Hence, mean dose parameters for the OARs are split into two groups based on the model's output values: when *P*(offVMAT) is less than 0.5 (Figure 4) and when *P*(offVMAT) exceeds 0.5 (Figure 6).

**FIGURE 5:**
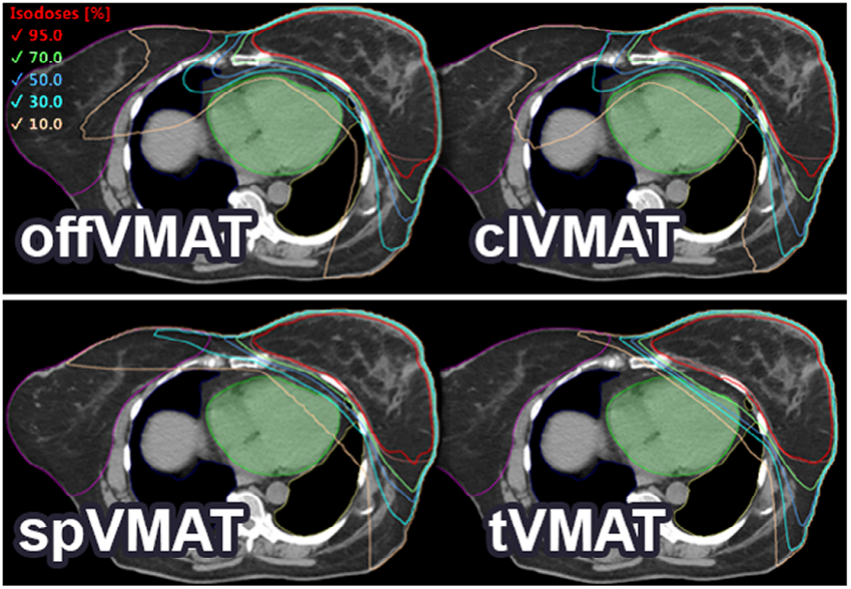
Example of isodose distribution of all techniques of patient with the parameter *P*(offVMAT) <0.5 (*P*_Heart_ = 0.07, *P*_Lung_ = 0.07 and *P*_Medial_ = 0.01) from training set on transverse views.

In patient group with *P*(offVMAT) < 0.5 (21 patients) both spVMAT and offVMAT techniques managed to adhere to all dose constraints. tVMAT failed in three instances concerning the ipsilateral lung, while clVMAT faltered in seven cases regarding the contralateral lung. (Figure 4). When *P*(offVMAT) < 0.5, tVMAT technique shows slightly higher doses, as in comparison with the offVMAT (V20, V10 of heart and V20, V10, and V5, mean dose of ipsilateral lung dose values); spVMAT shows better results in contralateral lung (V10, mean dose), but poorer results in contralateral breast (D2 and V5) in comparison with the offVMAT. Figures 4 and 5 demonstrates the large dose reductions of OAR in tVMAT compared with offVMAT, spVMAT and especially clVMAT in the dose parameters for contralateral organs. spVMAT shows intermediate results in mean dose parameters compared with tVMAT and offVMAT methods. clVMAT shows the worst results in contralateral organs and intermediate results between spVMAT and tVMAT in ipsilateral organs.

**FIGURE 6:**
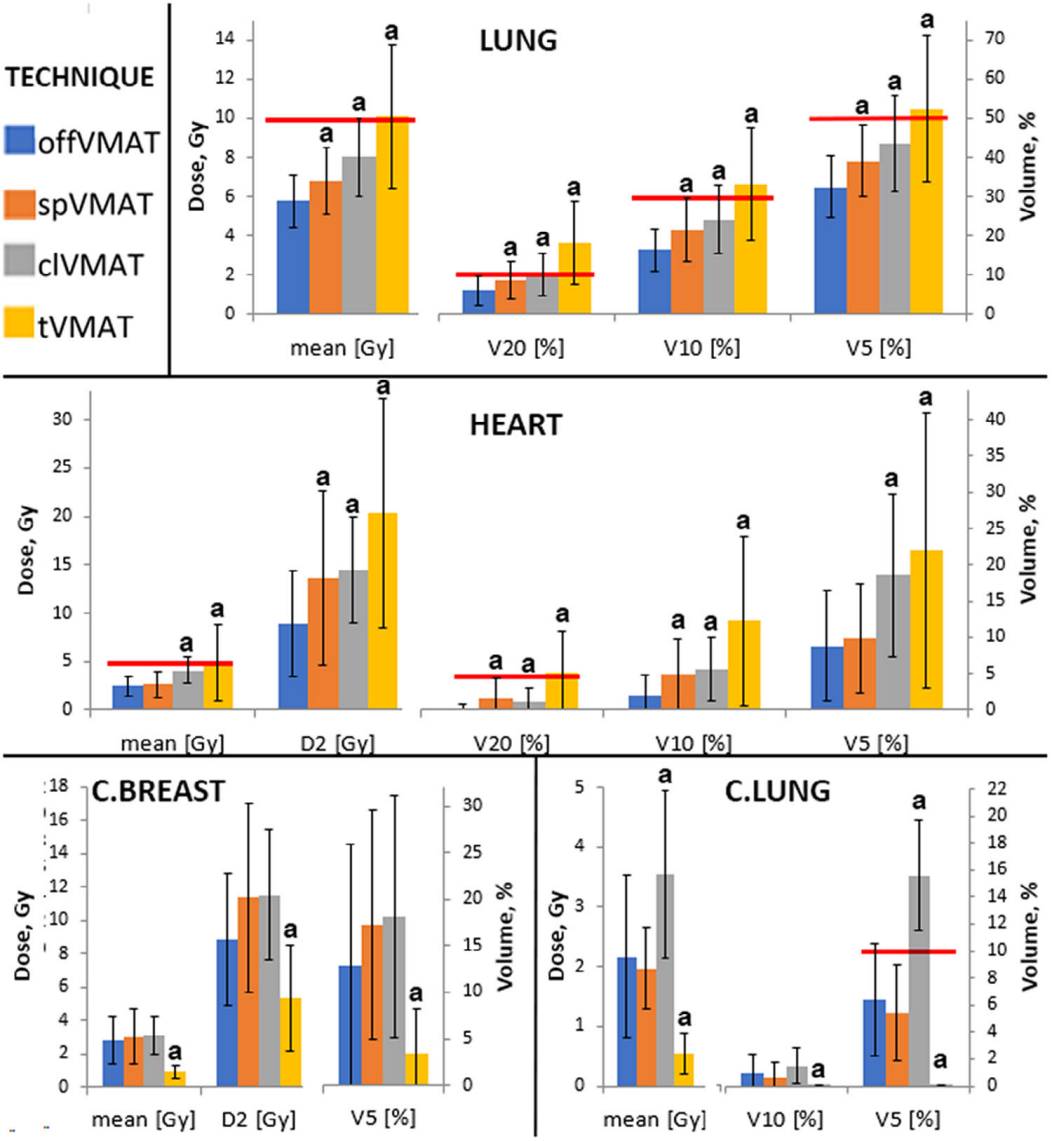
Dose parameters of organs at risk (OARs) (Mean ± SD) for patient group with *P*(offVMAT) >0.5 parameter (off‐isocentric volumetric modulated arc therapy [offVMAT], split volumetric modulated arc therapy [spVMAT], classic volumetric modulated arc therapy, [clVMAT], and tangential volumetric modulated arc therapy [tVMAT]). Red lines show the OAR constraint. ^a^Statistically significant difference (*p* < 0.05) in pairwise comparison against offVMAT technique.

The mean dose parameters for the OARs indicated in Figure 4 (*P*[offVMAT] < 0.5) and Figure 6 (P[offVMAT] > 0.5) were different between techniques.

In patient group with P(offVMAT) > 0.5 (23 patients), when using the offVMAT technique, one plan not meeting the heart constraint, four spVMAT plans failed lung and heart constraint, 13 tVMAT plans not adhering ipsilateral lung and heart constraint, and seven clVMAT plans failed contralateral lung and heart constraint (Figure 6). When *P*(offVMAT) > 0.5, the measured dose parameters for tVMAT methods are especially different from the values at *P*(offVMAT) < 0.5. For other techniques this trend is also present, but to a lesser extent. The Figure 6 shows that many tVMAT patients with the parameter *P*(offVMAT) > 0.5 are not able to achieve the planning goals: mean heart dose >5 Gy, and mean left lung dose >10 Gy. Although tVMAT still demonstrates the best dose distribution on the contralateral side among the techniques (Figure 6). clVMAT shows results worse than offVMAT for most criteria D2, V5 of contralateral breast, and V10 of contralateral lung. offVMAT compared with spVMAT shows lower dose values for V20, V10, and D2 of heart and for all planning goals of ipsilateral lung; there are also no advantages in dose distribution for contralateral OARs.

**FIGURE 7:**
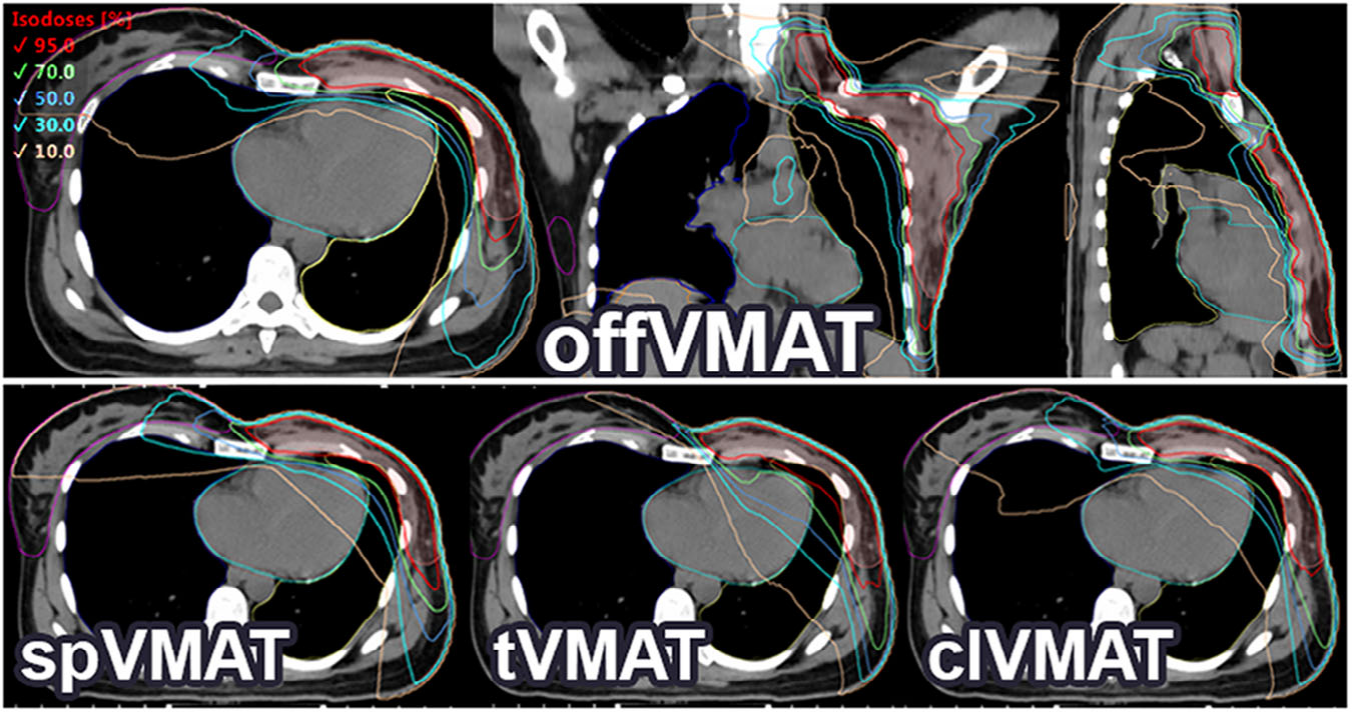
Isodose distribution of new off‐isocentric volumetric modulated arc therapy (offVMAT) technique of one of the most challenging patients with the parameter *P*(offVMAT) >0.5 (*P*_Heart_ = 0.26, *P*_Lung_ = 0.27 and *P*_Medial_ = 0.16) from training set on transverse, coronal, and sagittal views. Transverse views of other techniques are also presented for comparison.

The offVMAT technique, when *P*(offVMAT) leans toward 1, consistently demonstrated the lowest dose parameters for ipsilateral OARs while maintaining acceptable doses to the contralateral side (Figure 7).

All offVMAT plans passed QA using ArcCHECK (SunNuclear). Results showed a global gamma agreement of 98.1 ± 1.2% (range 96.6%−100.0%), implying that the off‐isocentric irradiation method is feasible for practical applications.

## 4 DISCUSSION

In this article, we introduce a new off‐isocentric VMAT technique (offVMAT) for breast treatment planning. During the application of this new technique, we found that it doesn't consistently yield the best results in achieving the dose criteria. Additionally, these results aren't contingent upon the levels of lymph nodes included in the PTV. The efficacy of a specific technique largely hinges on the patient's geometry. We've introduced anatomical parameters to provide a numerical representation of the patient's geometry, ensuring they are user‐friendly to save time during planning. Using these parameters, we constructed a regression model that delineates the applicability boundaries for the new offVMAT technique for breast cancer treatment.

### 4.1 Why does the off‐isocentric VMAT technique produce better outcomes compared to the classic VMAT?

The primary distinction between offVMAT and other techniques is the isocenter's displacement of 25–55 mm from the PTV boundary (or 25–75 mm from the PTV's center of mass) and simultaneously, the collimator jaw shifted over the central axis from the ipsilateral side by 2 cm—the maximum distance allowable for the X jaws. Because of this configuration, the primary beam is not directed at most times to the ipsilateral organs since it is always directed tangentially to the surface, with only scattered radiation delivering doses to ipsilateral OARs (Figure 2). Therefore, the new technique can potentially ensure enhanced coverage near the surface, crucial for irradiating thin chest walls. When the isocenter is positioned deeper into the lung (or heart), the dose to ipsilateral OARs diminishes, while the contralateral OARs' dose rises. For each patient, the isocenter's placement was decided based on the proximity and volume of the contralateral and ipsilateral OARs. The more significant the *P*_Heart_ and *P*_Lung_ values and the farther away the contralateral organs (especially on axial slices with heart presence), the more distant the isocenter was from the PTV. As such, the isocenter's position also serves as an optimized parameter. Authors of reference^19^ initially suggested shifting the isocenter outside the PTV by a precise 2 cm. However, based on our findings comparing offVMAT and spVMAT, 2 cm often isn't sufficient to achieve a drastic reduction in doses to ipsilateral OARs. Additionally, in the study,^19^ the isocenter wasn't artificially closed by the jaws, resulting in higher dose values in the ipsilateral organs. We also made attempts to bypass the 2 cm shift limitation over the central axis for the X‐jaw. To this end, a ∼90° collimator rotation was employed, ensuring the ipsilateral side was shielded with a Y‐jaw, which permits a 10 cm offset from the central axis. In such setups, the X‐jaws were also limited to less than 15 cm (often less than 7 cm, offset by an increased arc count). These configurations led to compromised PTV coverage and elevated doses to ipsilateral organs close to the PTV, attributable to the suboptimal positioning concerning the OARs and PTV during the gantry's movement.

At first glance, the task of OAR avoidance should be performed by the MLC and jaw tracking with the standard isocenter positioning (at the PTV's center of mass), for example, in clVMAT. However, positioning the isocenter farther from the PTV in the offVMAT technique reduces the required X‐jaw aperture size (Figure 2): 7.3 cm for offVMAT compared to 11.5 cm for clVMAT plans, averaged across all arc lengths and all patients. Consequently, the MLC needs to overcome a shorter average distance between the jaws in offVMAT. This can significantly influence the result due to the maximum speed limit of the leaves (2.5 cm/s for TrueBeam) and a more rapidly changing PTV projection (from a beam‐eye‐view) with the standard isocenter positioning.

Furthermore, the larger the field size with standard isocenter positioning, the greater the optimization parameter space, which can adversely affect the efficiency of finding the optimal local minimum. Also, the optimizer may allow small portions of the treatment beam to traverse the OARs (especially in gantry angles 0−90° for left breast treatment). With the off‐isocentric position, this effect is minimized due to the irradiation geometry (Figure 2a). Essentially, we assist the optimizer by setting the isocenter outside the PTV and pre‐limiting dosimetrically unfavorable irradiation angles with the X‐jaws. The optimization runtime for offVMAT was 10%−50% faster than for clVMAT for the same total arc lengths. However, the slightly greater complexity of arc arrangements (even when using plan templates) and also isocenter positioning in offVMAT might offset the overall speed of planning during the initial stages of implementing this technique. This can increase the planning process time by 2−3 times. However, the planner will not need to try different isocenter positions for most patients after gaining some experience (∼30–50 plans). Also, modifying the offVMAT arc template takes usually no more than 5 min. The Arc Geometry Tool (fine‐tune fields tab) can also help the planner in the Eclipse planning system at the initial stage. While the Fine‐tune Fields is open, it is possible to estimate the coverage on the target surface displayed in BEV or Model View before starting VMAT optimization. According to our experience, any part of the target should be irradiated at least 300° of gantry angles summed over all arcs to avoid issues with PTV coverage. The average time to create a plan was 44 ± 9 min, 29 ± 6 min, 37 ± 6 min, and 51 ± 8 min for offVMAT, tVMAT, spVMAT, and clVMAT respectively.

Additionally, in clVMAT, a significant portion of the low dose in OARs results from leaf transmission due to the increasing field size. In general, the more complex the PTV's shape, the more MLC modulation is needed, the more MUs are required to cover the target, and potentially, the more leakage passes through the MLC, especially without jaw tracking. Thus, the disadvantages of clVMAT only increase with shape complexity during breast irradiation. In contrast, in the offVMAT technique, only part of the target volume is inside the aperture, which isn't overlapped by ipsilateral OARs. The trade‐off is a larger number of MUs and beam‐on time, which is consistently observed when using the offVMAT technique (Table 3). The farther the isocenter is from the PTV, the more MUs are needed to cover the target. However, this doesn't increase the low dose to OARs via MLC leakage, since they are primarily shielded by the X‐jaws, not the MLC (Figure 2b).

### 4.2 Anatomical parameters and regression model

A broad spectrum of breast‐planning studies yields inconsistent conclusions across different publications due to various factors. These include differing clinical goals, planning experience of the physicist, the physician's experience, the use of DIBH,^8^, ^11^, ^29^ anatomical diversity from racial groups of patients,^13^, ^30^ and the type of guidance employed during the delineation of PTV and OARs.^31^, ^32^ By sorting and selecting patients for studies based on clinical parameters (like lymph node involvement), we can inadvertently introduce additional errors and distortions into the results. These distortions depend directly on the number of patients with specific anatomical characteristics, which may significantly influence the planning result. Within our random sample, there are patients with no nodal involvement, yet their anatomical parameters are comparable to those of patients with lymph node involvement. We believe, due to such inconsistencies, it's challenging to make objective comparisons between articles by different authors. However, this factor can be accounted for. It's essential to derive anatomical parameters that indicate the proximity and size of OARs to the PTV and ascertain the size of the PTV. Such parameters also make it harder to manipulate research results. Ideally, these parameters could be developed for other localizations as well. In this paper, we used anatomical parameters for transparent statistics and to determine the best VMAT technique for each case. We believe there's no single universal technique suitable for all patients (Figures 4, 5, 6, 7). As demonstrated in this article, anatomical parameters can be employed to decide which technique achieves lower dose levels to OARs before initiating breast planning. To construct such a model, we employed binary logistic regression (Equation 4).

The model (Equation 4), utilizing *P*_Heart_, *P*_Lung_, and *P*_Medial_ anatomical parameters, assesses the likelihood of achieving lower dose values to OARs among 24 patients when using the offVMAT as opposed to the not offVMAT technique. The variable *P*(offVMAT) approaches values near 0 (if not offVMAT is preferred) or 1 (if offVMAT is favored). Model (4) represents the relationships illustrated in the histogram (Figure 3): as the anatomical parameters *P*_Heart_ and *P*_Lung_ rise relative to *P*_Medial_, and their absolute values increase, so does the probability of opting for offVMAT.

Of course, this model is provisional and requires further refinement: it needs to be updated with more patient data, possibly introduce new anatomical parameters directly related to contralateral organs rather than through *P*_Medial_, be validated and trained on larger sample sizes, employ multinomial logistic regression for different techniques (not just VMAT) as more clinical cases accumulate, and possibly incorporate dosimetric plans from multiple planners and clinics to enhance the model's adaptability.

### 4.3 Advantages of off‐isocentric VMAT technique

Utilizing anatomical parameters and binary logistic regression, we delineated the boundaries of the new offVMAT technique's applicability. It's employed to achieve lower ipsilateral dose levels. The tVMAT and spVMAT techniques are suitable for patients with a *P*(offVMAT) < 0.5 (Figure 4). As observed in several studies,^10^, ^13^, ^14^, ^15^ tVMAT consistently demonstrates reduced doses to contralateral OARs (Figure 4). When *P*(offVMAT) < 0.5, spVMAT exhibits higher doses to the patient's contralateral side but compensates with reduced levels of medium and high doses to the heart compared to tVMAT. Dose parameters in the offVMAT technique show higher values for contralateral lung (mean, V10), and lower values for contralateral breast (D2 and V5) when compared to spVMAT. However, offVMAT ensures a slight decrease in high doses to ipsilateral OARs (Figures 4 and 5). For the patient group with *P*(offVMAT) < 0.5, the technique choice remains ambiguous since clinical objectives are met using any method. However, both tVMAT and spVMAT have their respective merits. The clinical plan should be selected based on the patient's medical history. If the paramount goal is to spare contralateral OARs or reduce beam‐on time (e.g., when using DIBH), then tVMAT is the optimal choice. Conversely, if the objective is to decrease high dose levels to ipsilateral OARs, the spVMAT technique prevails. The clVMAT, despite its prevalent use in clinical practice, doesn't exhibit any advantages. It consistently underperforms in all dosimetric criteria across all patient categories compared to other methods. In juxtaposition with various studies,^1^, ^15^, ^19^, ^20^ Figures 4 and 6 highlight comparable or reduced dose parameters for all OARs for tVMAT, spVMAT, and clVMAT techniques, recalculating the dose due to a 2 Gy, 25fr prescription. Nonetheless, drawing comparisons between dose values from disparate articles is challenging without knowledge of patient anatomical parameters.

For the patient group with *P*(offVMAT) > 0.5, the new offVMAT technique distinctly exhibits superior dose distribution (Figures 6 and 7). In offVMAT plans, the mean dose parameters for the heart, when compared to spVMAT, show V20, V10, and D2 are lower by factors of 7.1, 2.4, and 1.5 respectively (Figure 6). Achieving advantages in the ipsilateral lung using offVMAT is evident: V20, V10, V5, and mean dose are lower by factors of 1.4, 1.3, 1.2, and 1.2, respectively (Figure 6). The average doses to the contralateral side are consistent (Figure 6). In our view, when *P*(offVMAT) > 0.5, the tVMAT technique should be judiciously employed, given the potential risk of not meeting constraints on ipsilateral organs. The clVMAT also consistently demonstrates inferior results for most criteria when juxtaposed with offVMAT and spVMAT (Figure 6).

The proposed offVMAT technique and regression model (Equation 4) are being integrated into our clinic as standard practice. Moreover, our department has already successfully employed the off‐isocentric planning technique for other irradiation targets. Exploring a more precise relationship between all potential anatomical parameters and planning techniques is beneficial. It allows for the prediction of the best technique for each patient individually and aids in the development of auto‐planning software.

This manuscript's content and framework of this manuscript were constructed for consistency with the recently published RT treatment planning guidelines for generating high‐quality planning studies.^33^ The RATING score was calculated and equaled 97% (Supplementary Materials S02).

## 5 CONCLUSION

The article introduces a new off‐isocentric breast planning technique. While it can be used regardless of the level of lymph node involvement, it holds a distinct advantage over other techniques in cases of complex patient's geometry. Specifically, as the parameter *P*(offVMAT) approaches 1, offVMAT outperforms other techniques, demonstrating better dose values for V20, V10, D2 of the heart, and V20, V10, V5, as well as the mean dose of the ipsilateral lung, all while maintaining an acceptable dose to the contralateral side. Importantly, the optimal choice of technique largely depends on the specific patient's geometry. This selection can be refined using the patient anatomical parameters and the regression model proposed in the article, rather than merely relying on information about the involved lymph nodes. Furthermore, the utilization of anatomical parameters and the associated regression model not only enhances the accuracy and transparency of comparing planning techniques across various articles but can also aid in the development of auto‐planning.

## AUTHOR CONTRIBUTIONS

*Lead author of manuscript, data collection and analysis, regression modelling, and corresponding author*: Igor Prokofev. *Study design, data analysis, and clinical expertise*: Nidal Salim.

## CONFLICT OF INTEREST STATEMENT

The authors declare no conflicts of interest.

## ETHICAL APPROVAL

Ethical approval for this study was obtained by the Local Research Ethics Committee at the European Medical Center on March 11, 2021.

## Supporting information

Supporting Information

Supporting Information

## Data Availability

Data available on request from the authors
